# Supplementary material for: Isavuconazole Pharmacokinetics and Pharmacodynamics in Children
Source: Pharmaceutics. 2022 Dec 26;15(1):75. doi: 10.3390/pharmaceutics15010075 (PMC9865364; doi:10.3390/pharmaceutics15010075)
Supplement: Supplementary file 1 [file pharmaceutics-15-00075-s001.zip › pharmaceutics-2066078-supplementary.pdf]

pharmaceutics

Table S1. Model support points. Parameters are as defined in text and in Table 1.

Ke0 (h^{-1})	V0 (L)	Ka (h^{-1})	KCP0 (h^{-1})	KPC0 (h^{-1})	Probability
1.5×10^{-05}	47.50	12.00	6.38	0.50	0.10
0.12	50.13	12.00	0.10	0.03	0.21
0.12	40.88	12.00	2.42	0.23	0.24
0.04	73.32	0.01	2.34	5.00	0.06
0.10	37.27	0.04	4.85	2.49	0.08
0.05	57.71	0.02	4.39	0.78	0.17
0.09	24.60	1.08	0.18	1.34	0.09
0.09	24.58	1.08	0.18	1.34	0.05

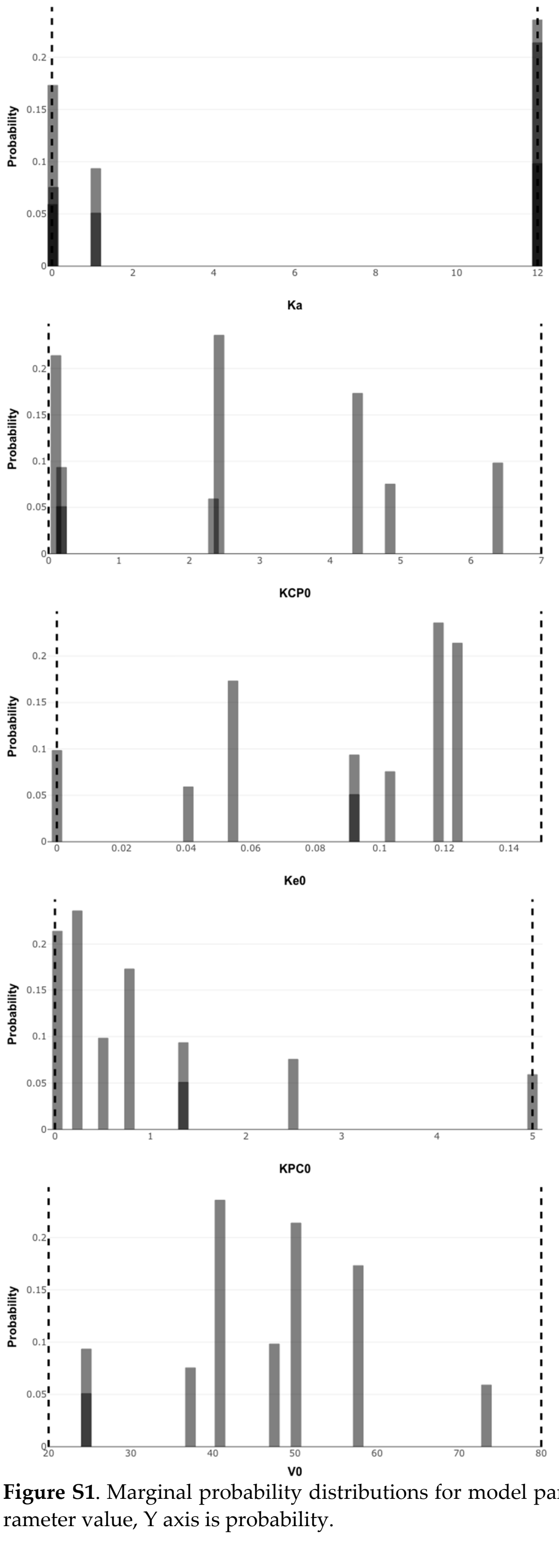

Figure S1. Marginal probability distributions for model parameter values. Vertical dashed lines are fixed boundaries. X axis is parameter value, Y axis is probability.

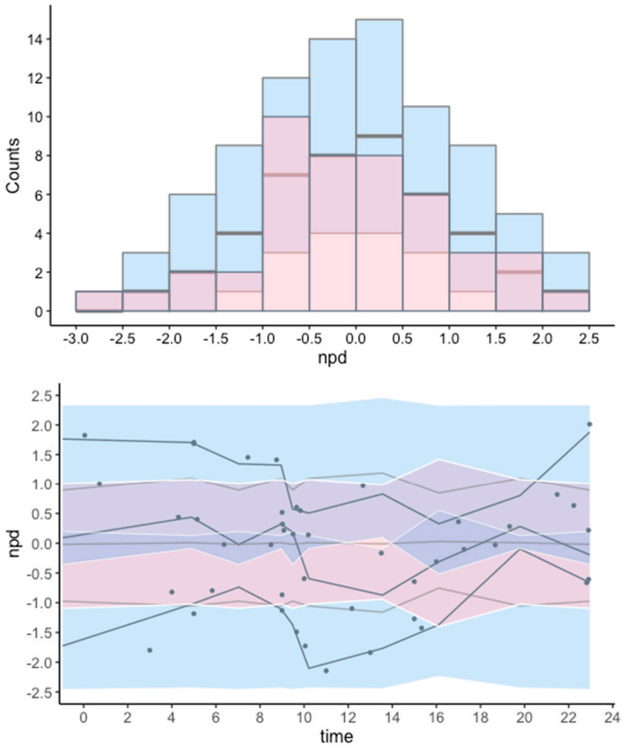

Figure S2. Normalized prediction distribution error (npde). Top left shows histogram of theoretical npd distribution according to normal distribution (blue) with mean and 95% CI, and model distribution (pink) within the expected distribution. Top right is Q-Q plot with theoretical (grey), compared to model derived line (dark grey), entirely within the 95% CI for expected distribution (blue). Bottom row shows time (left) and predicted (right) vs. npd. There is considerable overlap, but upper light grey line is the 95%ile of simulated concentrations. The upper darker line is the 95%ile of observations, lying within the upper blue region, which is the 95% CI surrounding the 95%ile of simulated concentrations. The pink 95% CI region surrounds the median of simulations, and the lower blue surrounds the 5%ile of simulations. Observation percentiles are within the expected distributions by simulation in both plots, except for the small red region in lower right plot.
